# The role of GluN2A and GluN2B NMDA receptor subunits in AgRP and POMC neurons on body weight and glucose homeostasis

**DOI:** 10.1016/j.molmet.2015.06.010

**Published:** 2015-07-06

**Authors:** Aykut Üner, Gabriel H.M. Gonçalves, Wenjing Li, Matheus Porceban, Nicole Caron, Milena Schönke, Eric Delpire, Kenji Sakimura, Christian Bjørbæk

**Affiliations:** 1 Department of Medicine, Division of Endocrinology and Metabolism, Beth Israel Deaconess Medical Center and Harvard Medical School, Boston, MA 02215, USA; 2 Department of Anesthesiology, Vanderbilt University Medical School, Nashville, TN 37232, USA; 3 Department of Cellular Neurobiology, Brain Research Institute, Niigata University, Niigata 951-8585, Japan

**Keywords:** AgRP, NMDAR, GluN2B, Metabolism, Glycemia, Leptin, AgRP, agouti-related peptide, POMC, pro-opiomelanocortin, NMDARs, N-methyl-d-aspartate receptors, *Lep*^*ob/ob*^ mice, obese leptin-deficient mice, GABA, gamma-aminobutyric acid, EPSCs, excitatory post-synaptic synaptic currents, AMPARs, α-amino-3-hydroxy-5-methyl-4-isoxazolepropionic acid receptors, PVN, paraventricular nucleus, LTP, long-term potentiation, LTD, long-term depression, GTT, glucose tolerance test, ITT, insulin tolerance test, hrGFP, humanized renilla GFP, DAB, 3,3′-diaminobenzidine, DREADD, Designer Receptor Exclusively Activated by Dedigner Drugs, PBS, phosphate-buffered saline, RT, room temperature, ANOVA, analysis of variance, ANCOVA, analysis of covariance, HSD, honestly significant difference, KO, knockout, DIO, diet-induced obesity, HFD, high-fat diet, CNS, central nervous system, AUC, area under the curve, AAC, area above the curve

## Abstract

**Objective:**

Hypothalamic agouti-related peptide (AgRP) and pro-opiomelanocortin (POMC) expressing neurons play critical roles in control of energy balance. Glutamatergic input via n-methyl-d-aspartate receptors (NMDARs) is pivotal for regulation of neuronal activity and is required in AgRP neurons for normal body weight homeostasis. NMDARs typically consist of the obligatory GluN1 subunit and different GluN2 subunits, the latter exerting crucial differential effects on channel activity and neuronal function. Currently, the role of specific GluN2 subunits in AgRP and POMC neurons on whole body energy and glucose balance is unknown.

**Methods:**

We used the cre-lox system to genetically delete GluN2A or GluN2B only from AgRP or POMC neurons in mice. Mice were then subjected to metabolic analyses and assessment of AgRP and POMC neuronal function through morphological studies.

**Results:**

We show that loss of GluN2B from AgRP neurons reduces body weight, fat mass, and food intake, whereas GluN2B in POMC neurons is not required for normal energy balance control. GluN2A subunits in either AgRP or POMC neurons are not required for regulation of body weight. Deletion of GluN2B reduces the number of AgRP neurons and decreases their dendritic length. In addition, loss of GluN2B in AgRP neurons of the morbidly obese and severely diabetic leptin-deficient *Lep*^*ob/ob*^ mice does not affect body weight and food intake but, remarkably, leads to full correction of hyperglycemia. *Lep*^*ob/ob*^ mice lacking GluN2B in AgRP neurons are also more sensitive to leptin's anti-obesity actions.

**Conclusions:**

GluN2B-containing NMDA receptors in AgRP neurons play a critical role in central control of body weight homeostasis and blood glucose balance via mechanisms that likely involve regulation of AgRP neuronal survival and structure, and modulation of hypothalamic leptin action.

## Highlights

• Glutamate action via GluN2B in AgRP neurons is required for normal body weight.
• Genetic loss of GluN2B selectively in AgRP neurons reduces food intake.
• Deletion of GluN2B from AgRP neurons prevents diabetes in *ob/ob* mice.
• Glutamate action via GluN2B-NMDARs in AgRP neurons regulates neuronal survival and dendritic plasticity.
• GluN2B-NMDARs in AgRP neurons are novel anti-obesity and anti-diabetes drug targets.

## 1 Introduction

Hypothalamic agouti related peptide (AgRP) and proopiomelanocortin (POMC) producing neurons play important roles in the regulation of body weight homeostasis. Activation of AgRP neurons in mice promotes a state of positive energy balance by increasing energy intake and decreasing energy expenditure, while stimulation of POMC neurons promote a state of negative energy balance [1–6]. Arcuate nucleus (ARC) AgRP and POMC neurons are regulated by neuronal synaptic inputs, circulating metabolites and peripheral hormonal signals such as leptin, insulin and ghrelin. Both groups of cells also have the capacity to mediate potent anti-diabetic actions by leptin [7–9]. Although significant progress has been made in understanding the action of these hormonal signals in control of AgRP and POMC neurons on energy balance regulation [10–12], little is known about the roles of upstream neural inputs such as glutamate, gamma-aminobutyric acid (GABA) and serotonin [13–16].

N-methyl-d-aspartate receptors (NMDARs) are ligand-gated ion (Ca^2+^) channels activated by the neurotransmitter glutamate and are amongst the most important proteins in the post-synaptic density of excitatory synapses [17,18]. The NMDAR is a heteromeric channel comprised of two obligatory GluN1 (NR1) subunits together with two GluN2 subunits (GluN2A, 2B, 2C, or 2D) (NR2A-D), and/or GluN3 subunits (NR3A and NR3B). Most native NMDARs appear to function as assemblies composed of two GluN1 subunits and two GluN2 subunits, typically GluN2A and GluN2B [17,19]. The identity of the GluN2 subunits is critical for the biophysical and pharmacological properties of the receptor and for the regulation of neuronal processes. For example, GluN2B-containing NMDARs have longer decay times compared with GluN2A, thus integrating excitatory post-synaptic synaptic currents (EPSCs) across broader time intervals [20]. Also, GluN2A and GluN2B containing channels differentially control synaptic activity via distinct effects on translocation of α-amino-3-hydroxy-5-methyl-4-isoxazolepropionic acid receptors (AMPARs) to the post synaptic membrane [21]. Genetic loss of GluN2B in mice causes defective synaptic development and early postnatal death [22–24] while global deletion of GluN2A allows survival but causes impaired spatial learning [25].

Hypothalamic NMDARs can influence energy balance acutely as intrahypothalamic injection of glutamate analogs (NMDAR-specific) elicits robust eating behavior [26] whereas NMDAR-specific antagonists suppress feeding [27,28]. In addition, genetic deletion of the obligatory GluN1 subunit only from AgRP neurons reduces body weight and food intake [14]. Moreover, an excitatory paraventricular hypothalamic nucleus (PVN) to AgRP neuronal circuit that drives hunger has recently been described, further underscoring the importance of glutamatergic input to specific arcuate hypothalamic neurons in control of energy balance regulation [29]. While these data show that hypothalamic glutamatergic action and NMDARs are important for energy balance control in a neuron specific manner, the contributions of individual GluN2 subunits, specifically GluN2A and GluN2B, within the key AgRP and POMC neurons are unknown.

NMDARs mediate acute excitatory neurotransmission [30–32]. In the longer term, NMDARs also serve to regulate dendritic growth, arborization and spine density, thus influencing neuronal function and connectivity as well as neuronal development and cell survival [25,33–35]. In the hippocampus, these cellular events greatly depend on specific GluN2 subunits and underlie important processes such long-term potentiation and depression (LTP and LTD), which likely controls cognitive functions such as learning and memory [25,36]. In AgRP neurons, NMDARs regulate spine density and are required for normal responses to fasting [14]. Neuronal plasticity of POMC and AgRP neurons may also be critical for the regulation of energy homeostasis by the fat-derived hormone leptin [37,38].

Investigation of NMDARs and specific GluN2 subunits within critical hypothalamic neurons is therefore important for our full understanding of normal energy balance regulation and has the potential for gaining insights into novel anti-obesity drug targets.

## 2 Materials and methods

### 2.1 Animal studies and metabolic parameters

All studies were approved by the Beth Israel Deaconess Medical Center Institutional Animal Care and Use Committee and conducted in accordance with federal laws and guidelines. Unless otherwise specified, mice 3–19 weeks of age were used and single-housed at 22–24 °C using a 12 h light/12 h dark cycle with *ad libitum* access to standard mouse chow (LabDiet) and water. Body weight, food intake and body composition measurements, determination of blood composition including ELISA assays, and leptin sensitivity (500 ng/h for 13 days), glucose and insulin tolerance test (GTT/ITT) and pair-feeding were performed as we have described earlier [7,8].

### 2.2 High-fat diet

At 4 weeks of age, groups of mice were subjected to high-fat diet (Research Diets) consisting of 60% fat by energy (kcal). Control mice were fed a low-fat diet (16.7% fat by energy [kcal], (standard chow)).

### 2.3 Deletion of GluN2A and GluN2B in AgRP and POMC neurons

We used the cre-lox system to delete GluN2A or GluN2B subunits of NMDARs in AgRP or POMC neurons. *AgRP-ires-cre* and *POMC-cre* mice were generated as described earlier [39–41]. *GluN2A*^*flox/flox*^ and *GluN2B*^*flox/flox*^ mice were obtained from Drs. Nicoll and Sakimura [21], and Dr. Delpire [33], respectively. *GluN2A*^*flox/flox*^ (or *GluN2B*^*flox/flox*^) mice were crossed with *AgRP-ires-cre* (or *POMC-cre*) mice in order to generate *GluN2A*(or *2B*)^*flox/flox*^;*AgRP-ires-cre* (or *POMC-cre*) mice. Control *GluN2A*(or *2B*)^*flox/flox*^ mice and *GluN2A*(or *2B*)^*flox/flox*^;*AgRP-ires-cre* (or *POMC-cre*) study mice were obtained by mating *GluN2A*(or *2B*)^*flox/flox*^ mice with *GluN2A*(or *2B)*^*flox/flox*^;*AgRP-ires-cre* (or *POMC-cre*) mice. All studies were performed on age- and sex-matched littermates from these breedings.

### 2.4 Hypothalamic AgRP and POMC neuronal counting

Control and AgRP-GluN2B KO mice expressing human renilla (hr)GFP in AgRP neurons were generated by crossing *GluN2B*^*flox*/*flox*^, *AgRP*-*ires*-*cre* and *NPY-hrGFP* mice [42]. Similarly, control and POMC-GluN2B KO mice expressing hrGFP in POMC neurons were generated by crossing *GluN2B*^*flox*/*flox*^, *POMC*-*cre* and *POMC-hrGFP* mice [43].

Male mice at 8 weeks of age were deeply anesthetized with ketamin (40 mg/kg) and xylazine (10 mg/kg) and brains were fixated with formalin through transcardiac perfusion. Coronal brain sections were generated (4 series, 30 μm thickness) as described earlier [7,8]. To quantify the number of hypothalamic AgRP and POMC neuronal cell bodies, brain sections were immunostained for hrGFP as we have generally described earlier [8,44]. Briefly, one of the four series was incubated with hrGFP antibodies (Agilent Technologies) (1:2000). After washing, the slices were incubated with secondary antibodies (donkey-anti-rabbit), developed with a 3-3′-Diaminobenzidine (DAB) mixture (Vector Laboratories), and finally mounted onto superfrost plus microscope slides (VWR Scientific). The number of neurons in the arcuate hypothalamic nucleus was counted manually in all sections with positive cells (8–10 sections/series) under a light microscope (Olympus (40× or 100× objective and 10× ocular)). The combined cell count from all sections and both hemispheres were finally multiplied with four (1:4 series) to estimate the total number of hrGFP positive cells in the brain.

### 2.5 Dendritic morphology

Cre-Dependent rAAV8/hSyn-DIO-mCherry (3.8 × 10e12 virus molecules/mL) kindly provided from Dr. Bryan Roth of the University of North Carolina Vector Core was diluted 1:100 with PBS and injected bilaterally (100 nL each) into the arcuate nucleus (coordinates, bregma: anterior-posterior, −1.40 mm; dorsal-ventral, −5.75 mm; lateral, ±0.30 mm) of 5 weeks old *GluN2B*^*flox/flox*^;*AgRP-ires-cre* or *AgRP-ires-cre* (control) mice by using a stereotaxic system from KOPF instruments. This low concentration of virus was selected after testing several dilutions such that a total of only 5–10 neurons were expressing mCherry in the entire hypothalamus. This very low number of fluorescent neurons allowed for clear identification and analyses of individual soma and their associated fibers. After 3 weeks, animals were perfused as described above and brains cut in 150 μm thick coronal sections. Sections were washed three times, 10 min/each in PBS, pH 7.4, and permeabilized with 1 × PBS/0.4% Triton x100 (Fisher Biotech) for 1 h at room temperature (RT). A buffer with 1% bovine serum albumin (Sigma–Aldrich, Inc) and 3% normal donkey serum (Jackson ImmunoResearch Laboratories) in 1 × PBS/0.4% Triton ×100 was used to block the tissue for 1 h at RT. Sections were then incubated overnight with rabbit anti-DsRed primary antibody (Clonetech; 1:1000) in the blocking buffer. Next day, sections were washed with 1 × PBS/0.4% Triton ×100 three times, 10 min each and incubated for 2 h at RT with Alexa-594-anti-rabbit secondary antibody (Invitrogen; 1:500). Finally, the tissue was washed again in 1 × PBS/0.4% Triton x100 three times and mounted on slides with mounting medium (Vector Laboratories, Inc). A Carl Zeiss confocal microscope (Objective Plan-Apochromat 20×/0.8) was used to take serial Z-stacks of primary dendrites labeled with mCherry, and a 3D projection was generated with the LSM (Zeiss) software. The ImageJ software (NIH, v. 1.48s) was subsequently applied to measure dendritic lengths. The selection of neurons and their associated dendritic fibers for analyses were done as follows: 1) only somata located within the hypothalamic arcuate nucleus were selected for further analyses; 2) only fibers that were associated with identifiable arcuate somata were selected; 3) axonal fibers were excluded based on their small diameter relative to dendrites and/or presence of boutons: 4) only primary dendrites longer than 50 μm were measured (from soma to longest terminus); 5) dendrites that appeared to extend out of the brain section (severed during sectioning) were excluded from the study. AgRP neurons typically had 1–3 primary dendrites and few, if any branches.

### 2.6 Statistical analysis

All values are presented as means ± SEM. Repeated measures including body weight and food intake in normal-, pair- and high-fat diet, GTT and ITT, leptin sensitivity were tested by using repeated-measures 2-way analysis of variance (ANOVA) or analysis of covariance (ANCOVA) with Tukey HSD *post hoc* test for multiple comparisons and Student's t test or Mann–Whitney U test was done when comparing only two groups. ANCOVA was conducted for GTT and ITT, levels of glucose and insulin, and leptin sensitivity. For this purpose, body weight values were used as covariate even though body weight-matched values were obtained [45]. *Post hoc* analysis was performed when group differences and/or group-by-time interaction are significant by repeated-measures two-way ANOVA or ANCOVA. Logarithmic transformation was used for some data that have variances not homogeneous. P ≤ 0.05 were considered significant for all independent factors.

## 3 Results

### 3.1 Deletion of GluN2B in AgRP, but not in POMC neurons causes decreased body weight, caloric intake, fat mass, and circulating leptin levels

**Figure 1:**
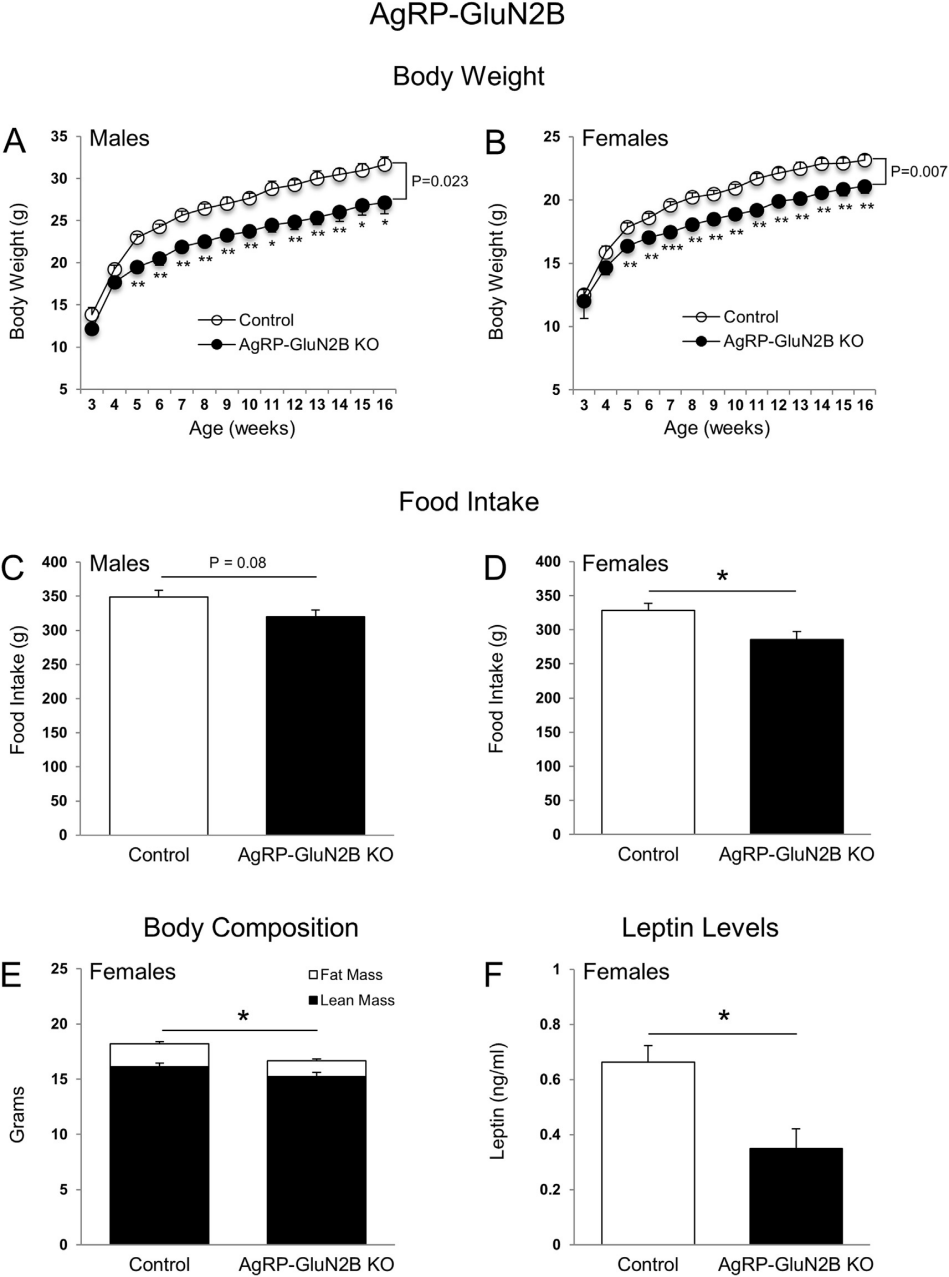
**GluN2B in AgRP neurons is required for normal body weight and food intake**. (A–D) Body weight (males (A) and females (B)) and cumulative food intake (weeks 3–15) (males (C) and females (D)) of control mice (*GluN2B*^*flox/flox*^) and *AgRP-GluN2B* KO mice. (E and F) Body composition (16 weeks of age) (E) and blood leptin levels (F) of control (*GluN2B*^*flox/flox*^) and *AgRP-GluN2B* KO female mice. Data are shown as means ± SEM (n = 4–11/group). P values in the line graphs are expressed as differences between groups (Student's t test) at the end of the study (week 16). Repeated measures two-way ANOVA and *post hoc* Tukey HSD tests were conducted to evaluate effect of GluN2B deletion from AgRP neurons on body weight. *Post hoc* multiple comparisons were performed using Tukey HSD test. Cumulative food intake and body composition at week 15 and leptin levels at week 17 were evaluated with Student's t test. *P ≤ 0.05, **P ≤ 0.01, ***P ≤ 0.001.

**Figure 2:**
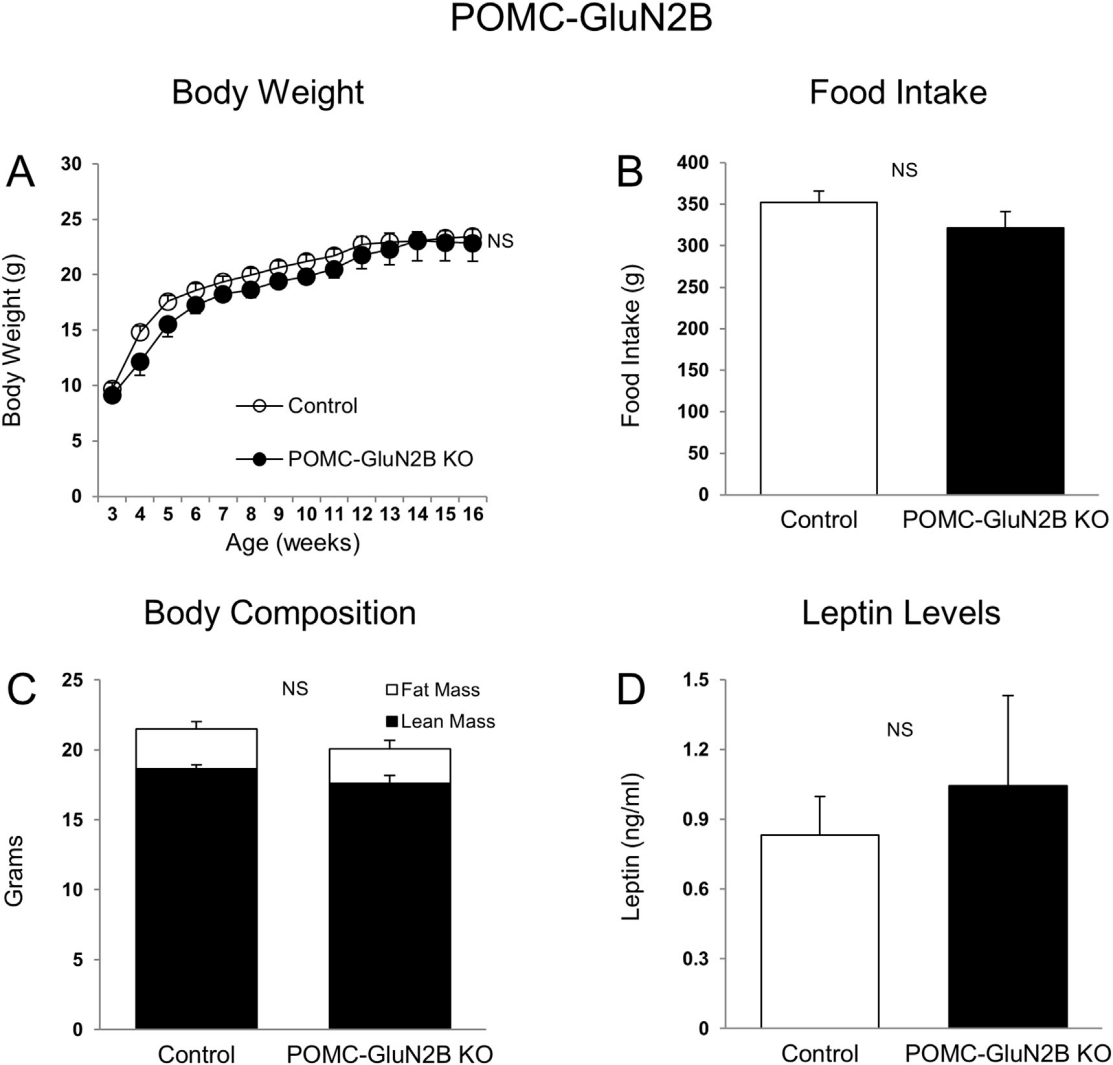
**GluN2B in POMC neurons is not required for normal regulation of body weight and food intake**. (A and B) Body weight (A) and cumulative food intake (weeks 3–15) (B) of control (*GluN2B*^*flox/flox*^) and *POMC-GluN2B* KO female mice. (C and D) Body composition (15 weeks of age) (C) and blood leptin levels (D) of control (*GluN2B*^*flox/flox*^) and *POMC-GluN2B* KO female mice. Data are shown as means ± SEM (n = 6–9/group). NS: Not significant.

**Figure 3:**
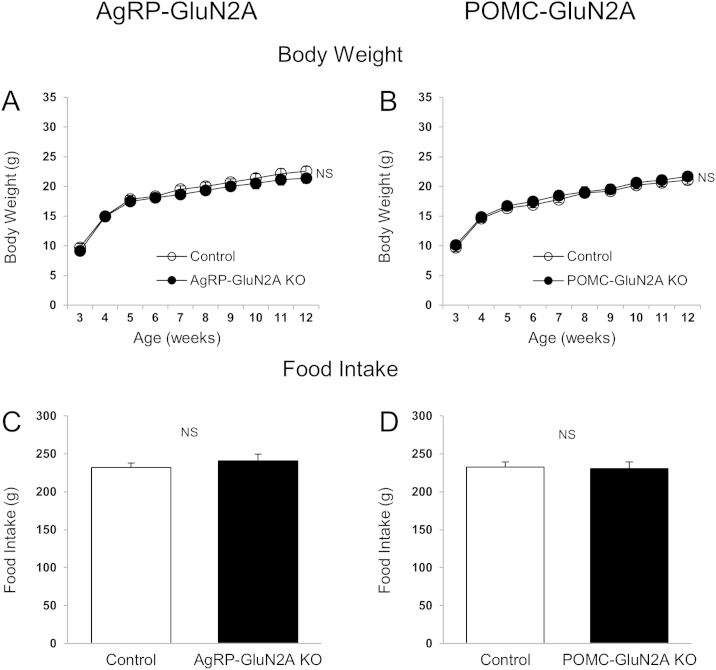
**GluN2A in AgRP and POMC neurons is not required for normal body weight and energy intake**. (A and C) Body weight (A) and cumulative food intake (C) (weeks 3–12) of control (*GluN2A*^*flox/flox*^) and *AgRP-GluN2A* KO female mice. (B and D) Body weight (B) and cumulative food intake (D) at week 12 of control (*GluN2A*^*flox/flox*^) and *POMC-GluN2A* KO female mice. Data are shown as means ± SEM (n = 7/group). NS: Not significant.

Intrahypothalamic injection of NMDAR/GluN2B agonists or antagonists elicits rapid feeding responses [26–28]. Furthermore, GluN1 deletion from AgRP neurons causes reductions in body weight, food intake, and body fat mass in mice [14]. Yet the role of specific GluN2 subunits within key hypothalamic neuronal groups remains unknown. We here examined whether GluN2A and GluN2B subunits in AgRP and POMC neurons are important for energy homeostasis control. We observed that mice lacking GluN2B in AgRP neurons had reduced body weight (Figure 1A and B), and cumulative food intake (Figure 1C and D) in male and female mice. Fat mass (Figure 1E) and blood leptin levels (Figure 1F) were also decreased in female mice. Male AgRP-GluN2B KO mice also exhibited lower fat mass relative to controls (2.75 ± 0.68 g; 4.66 ± 0.61 g; P = 0.07), although not quiet reaching statistical significance. In contrast, GluN2B deletion in POMC neurons did not affect body weight, food intake, body composition or leptin levels (Figure 2A–D). These results suggest that NMDAR GluN2B subunits in AgRP neurons are required for normal regulation of body weight homeostasis. In contrast, GluN2A neuron specific deletion from AgRP or POMC neurons did not affect body weight and food intake (Figure 3A–D). We therefore did not proceed with further studies of mice lacking GluN2A.

### 3.2 Pair-feeding and high-fat diet

**Figure 4:**
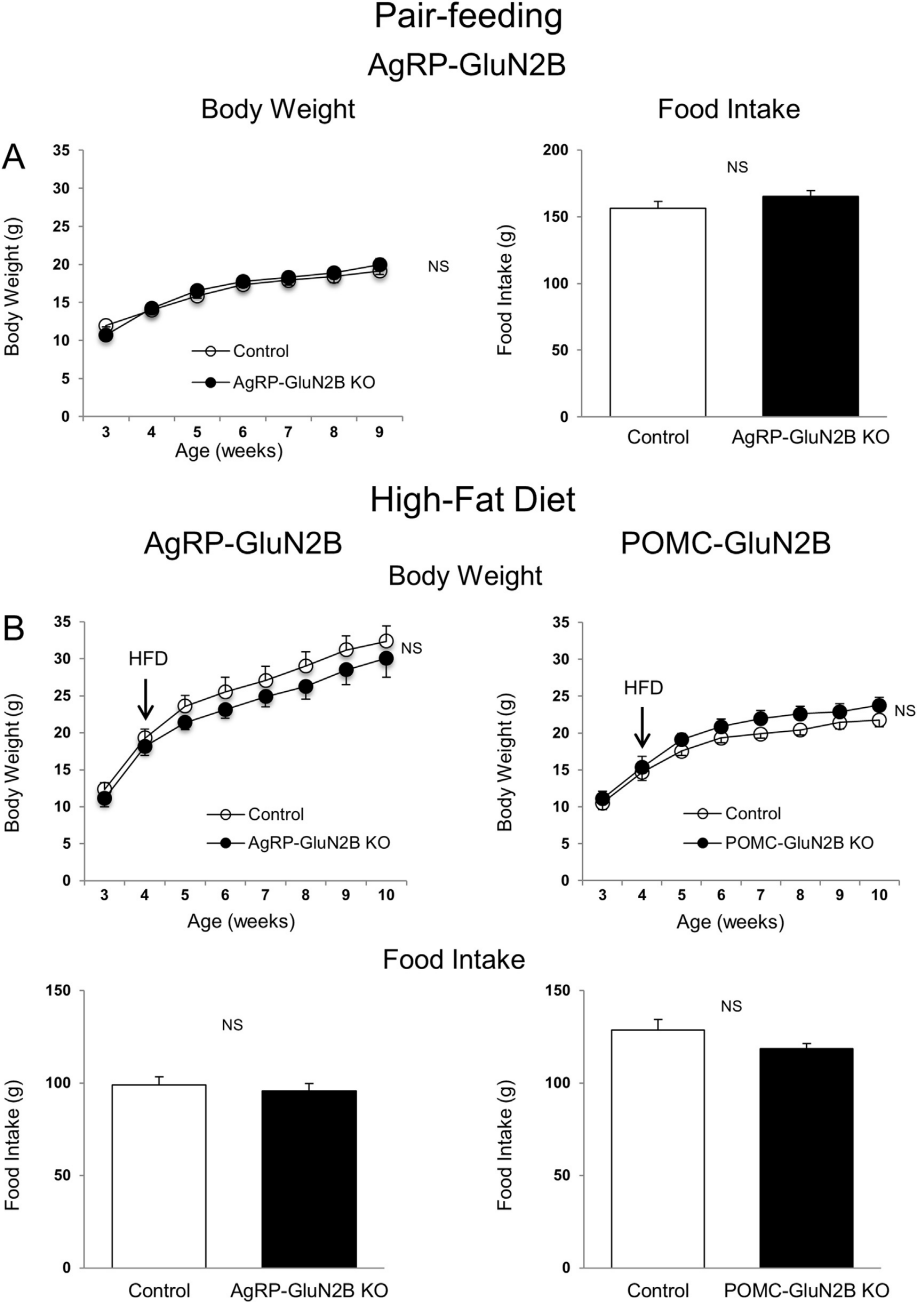
**Deletion of GluN2B in AgRP and POMC neurons**: **Pair-feeding and high-fat diet studies**. (A) Body weight and cumulative food intake (weeks 3–9) of control (*GluN2B*^*flox/flox*^) and *AgRP-GluN2B* KO female mice (n = 4–6/group). (B) Body weight and cumulative food intake (weeks 4–10) of control (*GluN2B*^*flox/flox*^) and *AgRP-GluN2B* KO male mice and control (*GluN2B*^*flox/flox*^) and *POMC-GluN2B* KO female mice during high-fat diet feeding. Data are shown as means ± SEM (n = 4–11/group). Repeated measures two-way ANOVA or Student's t test were conducted to evaluate differences between groups. NS: Not significant.

Because *GluN2B*^*flox/flox*^;*AgRP-ires-cre* mice were lean, we performed a pair-feeding study to determine whether this lower body weight was caused by reduced caloric intake or if increased energy expenditure might also be involved. Pair-feeding of control animals to the average intake level of *GluN2B*^*flox/flox*^;*AgRP-ires-cre* mice resulted in equal body weights between the two groups (Figure 4A), demonstrating that the reduced body weight of KO mice compared to *ad lib* fed controls (Figure 1A and B) is likely caused entirely by the reduction in caloric intake [45].

Diet-induced obesity (DIO) is characterized by hyperleptinemia, resistance to the anorexigenic effects of exogenous leptin, and by impaired leptin-receptor signaling within hypothalamic POMC and AgRP neurons [9]. In addition, AgRP neurons of DIO mice exhibit persistent activation and failure of leptin to reduce firing rates [46]. Since NMDARs regulate neuronal activity, we challenged wild type and KO mice with a high-fat diet (HFD) to investigate whether GluN2B in AgRP (and POMC) neurons influences sensitivity to develop DIO. Interestingly, the body weights and food intake of *GluN2B*^*flox/flox*^;*AgRP-ires-cre* mice on the HFD did not differ when compared to those of controls (Figure 4B), thus contrasting the lean and hypophagic phenotype of *GluN2B*^*flox/flox*^;*AgRP-ires-cre* mice relative to controls when fed the chow diet (Figure 1). Therefore, when exposed to HFDs, AgRP-GluN2B KO mice appear more susceptible to weight gain relative to control animals, if their lower base line body weight on chow diet is taken into consideration.

### 3.3 Deletion of GluN2B in AgRP and POMC neurons does not affect blood glucose, insulin levels, glucose tolerance and insulin tolerance

**Figure 5:**
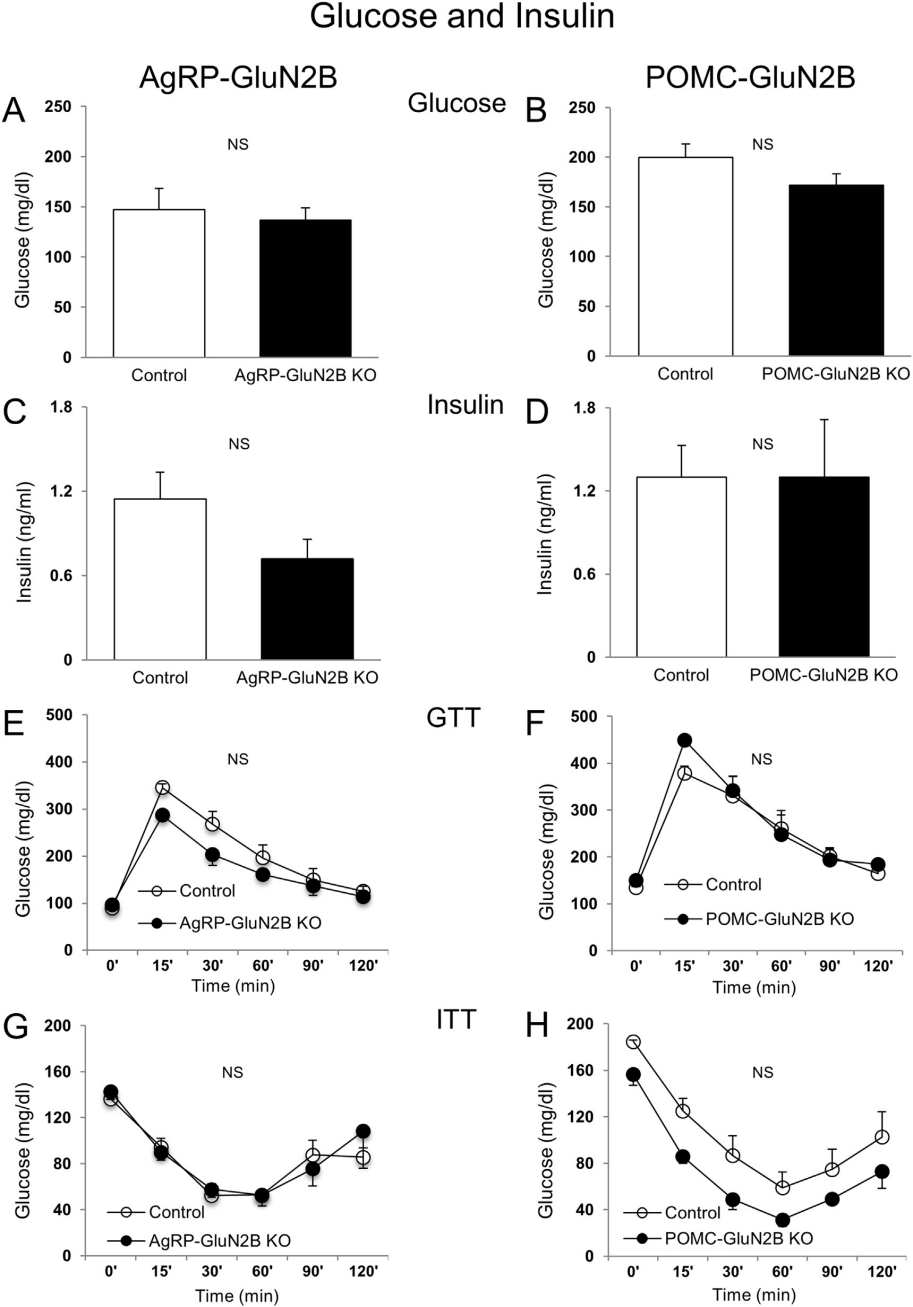
**Deletion of GluN2B in AgRP and POMC neurons: Blood glucose and insulin, and glucose- and insulin-tolerance**. (A–D) Fed blood glucose and insulin levels (15–17 weeks of age) in animals from AgRP (A and C) and POMC (B and D) studies, respectively. (E–H) Glucose tolerance test (GTT) following 15 h food removal (2 mg/g d-glucose, intraperitoneal) and insulin tolerance test (ITT) following 5 h food removal (1.2 U/kg insulin, intraperitoneal) (15–17 weeks of age) of AgRP (E and G) and POMC (F and H) studies, respectively. AgRP and POMC data are from female and male mice, respectively, and are shown as means ± SEM (n = 3–9/group). P > 0.05 for all experiments applying ANCOVA or repeated measures two-way ANCOVA. Logarithmic transformations were done for GTT values of AgRP study and ITT values of POMC study. Controls represent *GluN2B*^*flox/flox*^ mice. NS: Not significant.

Because AgRP and POMC neurons can respond to glucose and orchestrate blood glucose control [7,13,43,47], we measured fed glucose levels and performed glucose (GTT) and insulin (ITT) tolerance tests in young body-weight matched mice. GluN2B deletion in AgRP and POMC neurons did not affect fed blood glucose or serum insulin levels (Figure 5A–D), GTTs (AUC [AgRP]: 20.2 ± 0.76 × 10^3^ and 24.1 ± 1.48 × 10^3^ AgRP-GluN2B KO vs control, P > 0.05. AUC [POMC]: 31.6 ± 2.63 × 10^3^ and 30.5 ± 1.40 × 10^3^ POMC-GluN2B KO vs control, P > 0.05) (Figure 5E and F) or ITTs (AAC [AgRP]: 9.19 ± 0.97 × 10^3^ and 9.12 ± 0.71 × 10^3^ AgRP-GluN2B KO vs control, P > 0.05. AAC [POMC]: 7.46 ± 0.84 × 10^3^ and 10.8 ± 1.60 × 10^3^ POMC-GluN2B KO vs control, P > 0.05) (Figure 5G and H).

### 3.4 Elimination of GluN2B in AgRP neurons of diabetic and obese *Lep*^*ob/ob*^ mice normalizes hyperglycemia and increases leptin sensitivity

**Figure 6:**
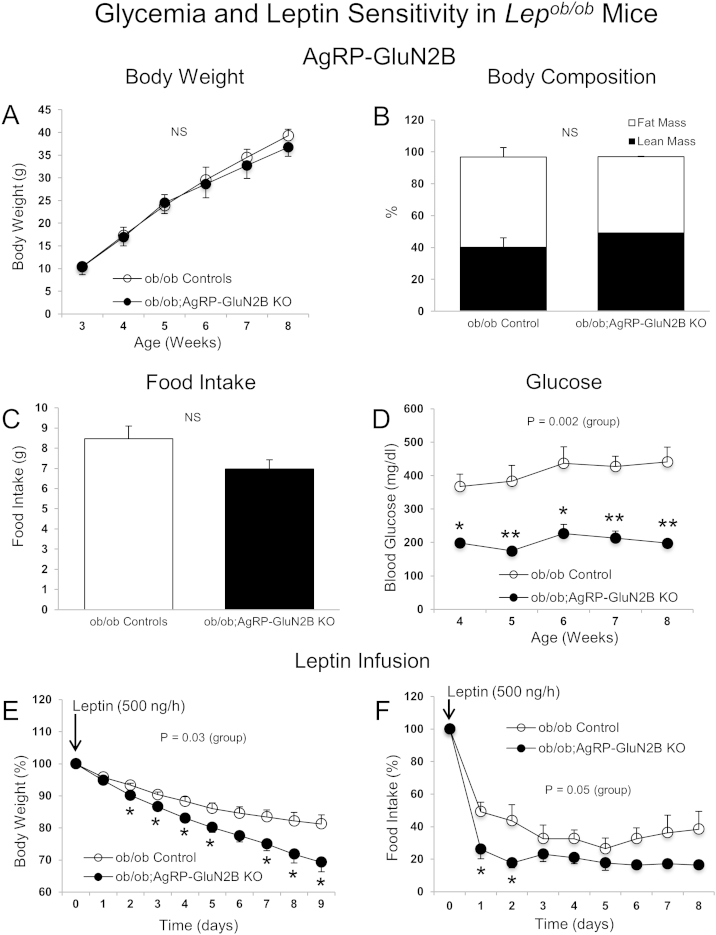
**Elimination of GluN2B in AgRP neurons of *Lep***^***ob/ob***^**mice normalizes hyperglycemia independently body weight**. (A–D) Body weight (A), body composition (8 weeks of age) (B), average daily food intake (week 7–8) (C), and blood glucose (D) in *ob/ob* controls (*ob/ob;AgRP-ires-cre* and *ob/ob;GluN2B*^*flox/flox*^) and *ob/ob;AgRP-GluN2B* KO female mice. (E and F) Body weight (E) and food intake (F) of female mice (9 weeks of age) after implantation of osmotic pumps loaded with leptin (500 ng/h) (9 days). Values in E and F are shown as percent of initial body weight and food intake, respectively. Data are shown as means ± SEM (n = 3–12 mice/group). Repeated measures two-way ANOVA or Student's t test were conducted to evaluate differences between groups. *P ≤ 0.05, **P ≤ 0.01. NS: Not significant.

We have reported earlier that exogenous leptin can normalize hyperglycemia in diabetic, obese and leptin-deficient *Lep*^*ob/ob*^ mice (*ob/ob*) independently of changes in body weight and caloric intake [7]. This effect of leptin required presence of leptin receptors in AgRP neurons and an intact central melanocortin receptor system. Since NMDARs affect neuronal polarization and activity, we generated *ob/ob* mice lacking GluN2B subunits in AgRP neurons (*ob/ob;AgRP-GluN2B* KO) and examined body weight and glucose balance, and tested leptin sensitivity. We found that loss of GluN2B in *ob/ob* mice did not affect body weight, body composition and food intake (Figure 6A–C), contrasting the lean phenotype of non-*ob/ob* mice lacking GluN2B in AgRP neurons (Figure 1). Of additional interest, deletion of GluN2B from AgRP neurons in the diabetic *ob/ob* mice entirely normalized hyperglycemia (Figure 6D). Moreover, in response to exogenous leptin infusion over a 9-day period, *ob/ob* mice lacking GluN2B subunits in AgRP neurons lost more body weight and exhibited a further reduced food intake relative to *ob/ob* control animals (Figure 6E and F). Combined, these results show that impaired signaling through GluN2B-NMDARs in AgRP neurons of diabetic and obese mice causes a major improvement in blood glucose control and increases sensitivity to leptin's anti-obesity actions.

### 3.5 Deletion of GluN2B from AgRP neurons reduces neuronal cell number and alters dendritic morphology

**Figure 7:**
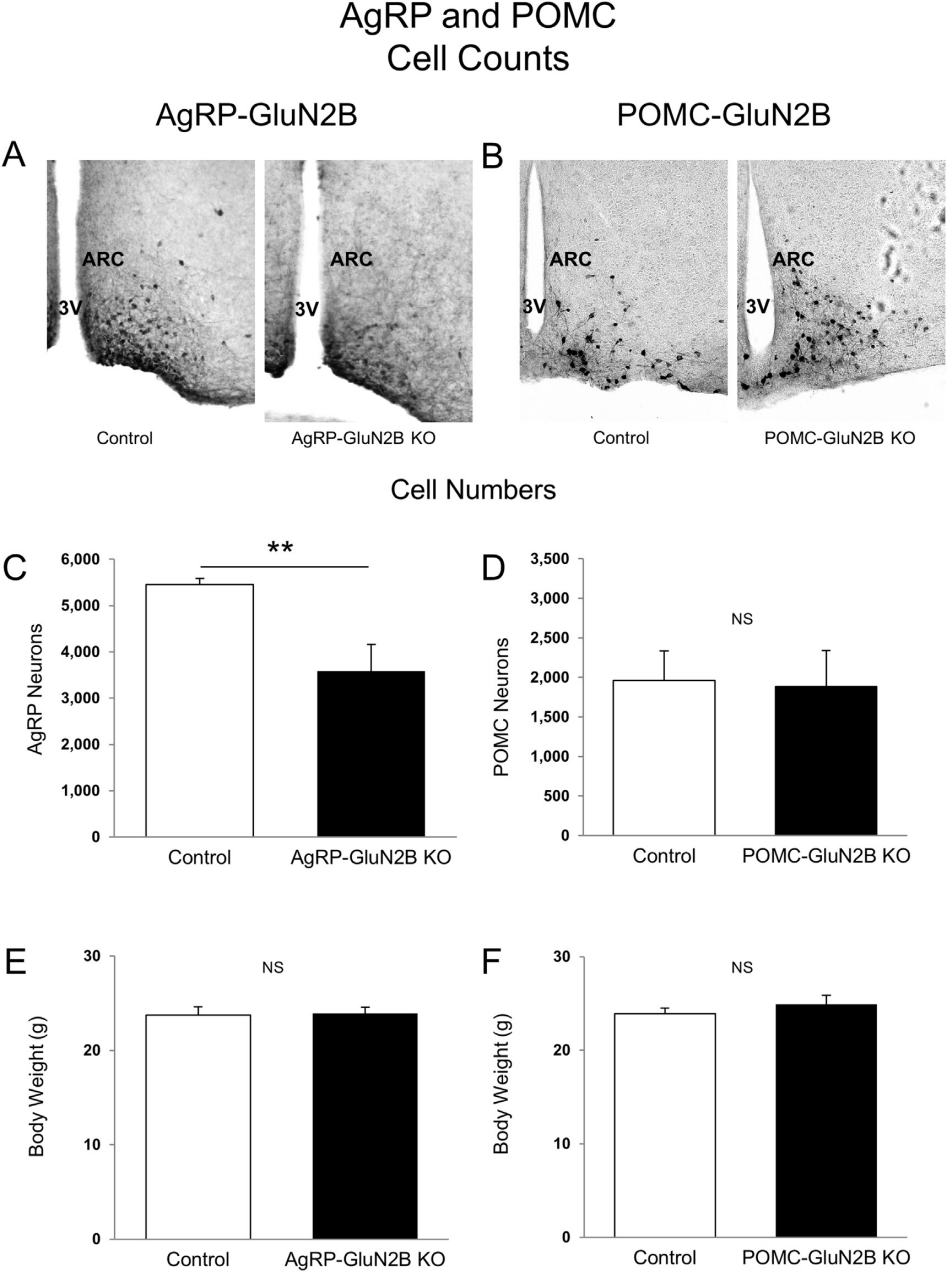
**Deletion of GluN2B from AgRP and POMC neurons: Neuron numbers** (A–F) Representative microphotographs (hrGFP immunohistochemistry) and cell counts of AgRP (A and C) and POMC (B and D) neurons in brain sections from body weight-matched (E and F) male control and KO animals (8 weeks of age). Data in Figure 8C and D are presented as the mean neuron number ± SEM (n = 4–5 mice/group). Differences between groups were evaluated with Student's t test. NS: Not significant. ARC: Arcuate nucleus. 3V: Third ventricle. Images in (A) and (B) were captured at 20× magnification.

**Figure 8:**
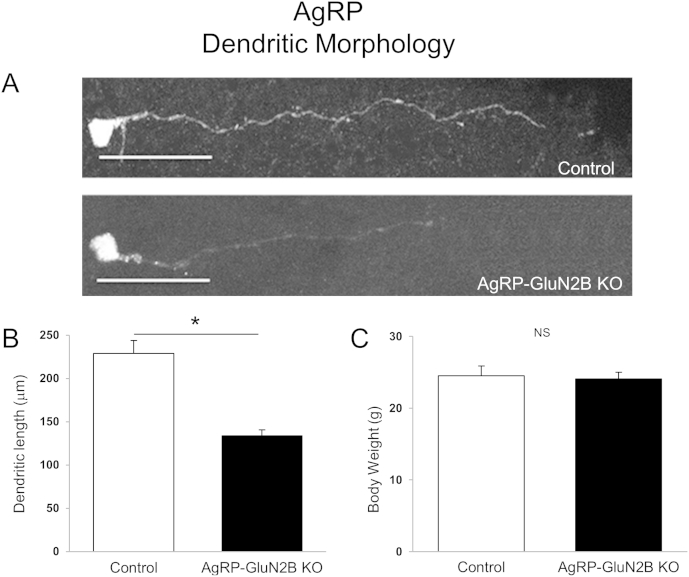
**Deletion of GluN2B from AgRP Neurons: Dendritic length**. (A) Representative fluorescence microphotograph (presented in grey tone) of dendrites of AgRP neurons in *AgRP-GluN2B KO* and control (*GluN2B*^*flox/flox*^) male mice at 8 weeks of age. (B) Dendritic length of *AgRP-GluN2B KO* and control (*GluN2B*^*flox/flox*^) male in 8 week old mice. (C) Body weight-matched groups of control and KO mice were pre-selected for these studies. Bar graph in Figure *B* shows the mean dendritic length ± SEM (n = 4–9 dendrites/group and n = 2–4 mice/group). *P ≤ 0.05, Student's t test between neuron-specific GluN2B-deleted mice versus control mice (*GluN2B*^*flox/flox*^) at week 8. Scale bar in (A) represents 50 μm.

NMDARs play a key role in neuron survival, neuronal plasticity and dendritic development, and even short-term blockade of NMDARs can trigger rapid neurodegeneration in the brain [35]. Therefore, since mice lacking GluN2B subunits in AgRP neurons have altered feeding behavior, we examined the number of AgRP neurons and assessed dendritic morphology in AgRP-GluN2B KO mice. To avoid potential indirect effects due to differences in body weight, body weight-matched KO and control mice were pre-selected for these studies (Figure 7E and F). We found that loss of GluN2B subunits from AgRP neurons caused a marked decrease (∼35%) in the number of AgRP neurons (Figure 7A and C). In contrast, deletion of GluN2B from POMC neurons did not affect hypothalamic POMC cell numbers (Figure 7B and D). In addition, GluN2B deletion from AgRP neurons resulted in a decrease (∼40%) in AgRP dendritic length (Figure 8A and B), also independently of body weight (Figure 8C). These data suggest that GluN2B NMDARs in hypothalamic AgRP neurons influence cell number and dendritic morphology. Therefore, the reduced body weight and energy intake of mice lacking GluN2B in AgRP neurons may be caused, at least in part by reduced neuronal survival and/or by altered dendritic morphology.

## 4 Discussion

The principal finding from these studies is that the NMDA receptor subunit GluN2B in hypothalamic AgRP neurons, but not POMC neurons, is required for normal body weight homeostasis in mice. In addition, loss of glutamatergic input via GluN2B-containing NMDARs expressed on AgRP neurons entirely prevents development of diabetes in obese leptin-deficient animals. Finally, NR2B-NMDARs control survival and dendritic morphology of AgRP neurons. These data may be relevant for future development of novel treatment therapies for human diabetes and obesity.

Hypothalamic AgRP and POMC neurons serve important functions in the regulation of energy balance through integration of metabolic, hormonal and neuronal inputs [1,7,13,43,47]. While significant progress has been made in our understanding of hormonal actions via these neurons, little is known about the importance of synaptic input by various neurotransmitters and action via their cognitive post-synaptic receptors. We here explored the roles of GluN2A and GluN2B, the two principal NMDAR glutamate-binding subunits [17], in AgRP or POMC neurons on metabolic and neuronal parameters in mice. Our results show that GluN2B in AgRP neurons is required for normal body weight homeostasis. In contrast, mice with loss of GluN2B expression in POMC neurons exhibit no body weight- or feeding intake-phenotype. We also show that genetic deletion of GluN2A in AgRP and POMC neurons does not affect these metabolic parameters. The GluN2B results are similar to data obtained from studies of mice with AgRP-specific deletion of the obligatory GluN1 NMDA receptor subunit [14]. These studies combined suggest that the principal functional NMDAR subunit-configuration expressed in hypothalamic AgRP neurons is GluN1/GluN2B.

It is unclear why deletion of GluN2B or GluN2A from POMC neurons does not influence body weight or feeding behavior. Clearly POMC neurons do express functional NMDARs as genetic deletion of GluN1 results in a complete loss of NMDAR-dependent EPSCs [14]. One simple explanation is that neither GluN2A nor GlutN2B are normally expressed in these cells, thus leaving possible roles of other GluN2 (or GluN3) subunits. Alternatively, because several subpopulations of hypothalamic and extra-hypothalamic POMC neurons are known to exist, including a hindbrain population [48–51], different cell groups may functionally oppose each other with regard to body weight regulation. Deletion from all POMC neurons will therefore result in an average phenotype. It is also conceivable that long-term loss of either GluN2B or GluN2A (or GluN1) is compensated during development by altered neuronal circuits or genetic redundancy. Further studies are needed to achieve understanding of this issue.

It is established that hypothalamic AgRP neurons can influence feeding behavior and body weight [1,3,4,52]. This is mediated in part by the action of circulating factors including the fat-derived hormone leptin, which informs these and other central nervous system (CNS) neurons about whole body energy storage in the form of adipose tissue [39,53,54]. We therefore investigated if the lean phenotype of GluN2B-AgRP KO mice might be caused, at least in part, by altered leptin action. We found that loss of GluN2B from AgRP neurons of leptin-deficient *Lep*^*ob/ob*^ mice did not result in a reduction in body weight, contrasting the data from *Lep*^*+/+*^;GluN2B-AgRP KO mice, suggesting that leptin is required for this latter phenotype. To examine this further, GluN2B-AgRP KO and control animals were given a high-fat diet (HFD) which normally leads to leptin resistance in hypothalamic neurons, accompanied by hyperphagia and accelerated weight gain [46,55,56]. Interestingly, GluN2B-AgRP KO mice exhibited the same caloric intake and growth curve as control mice, therefore differing from the lower body weight gain and hypophagia observed when KO animals were provided a low-fat-chow diet. Thus, in two models of impaired leptin action (*Lep*^*ob/ob*^ and diet-induced leptin resistance), the loss of GluN2B in AgRP neurons *does not* appear to influence body weight balance, altogether indicating that AgRP neurons normally serve as important nodes integrating glutamate and leptin signals to regulate feeding and body weight. Indeed, *Lep*^*ob/ob*^ mice lacking GluN2B only in AgRP neurons had increased sensitivity towards the anti-obesity actions of recombinant leptin suggesting that GluN2B-NMDARs normally antagonize leptin signaling within hypothalamic AgRP neurons. This is consistent with the notion that leptin inhibits AgRP neuronal activity [46], while glutamate typically increases neuronal activity.

These results raise several questions, including the nature of mechanisms explaining the lack of a lean phenotype of GluN2B-AgRP KO mice when given a HFD. One possibility is that the HFD, directly (via nutrients/factors in the diet) or indirectly (metabolites/hormones), causes AgRP neurons to reach maximal neuronal activity (e.g. firing) driving orexigenic pathways forward, regardless of the (presumed) lower baseline activity in GluN2B-AgRP KO mice due to reduced glutamatergic input and/or increased leptin sensitivity. This possibility could be tested with electrophysiological methods or by applying DREADD (Designer Receptor Exclusively Activated by Designer Drugs) technology. Alternatively, it is conceivable that the orexigenic capacities of AgRP neurons are negated in states of diet-induced obesity, either because of other neurons increasingly acting as the primary drivers of feeding and body weight or because of changes in neurocircuitry/plasticity. Indeed, ablation of AgRP neurons in neonates can be fully compensated, although to a lesser degree in adults [4,52].

In addition to its well described anti-obesity actions, leptin also has potent beneficial effects on blood glucose balance [9]. These anti-diabetic effects in Type 2 diabetic animal models are mediated to a large extent by inhibition of hypothalamic AgRP neurons and AgRP neuropeptide release, presumably involving disinhibition of the downstream melanocortin system [7,57]. Loss of glutamate action via GluN2B containing NMDARs in AgRP neurons of wild-type mice did not affect fed blood glucose levels, or insulin- or glucose-tolerance. Interestingly however, deletion of GluN2B from AgRP neurons of the severely diabetic and morbidly obese *Lep*^*ob/ob*^ mice completely prevented development of hyperglycemia, independently of changes in body weight and food intake. AgRP neurons in *Lep*^*ob/ob*^ mice are greatly depolarized and exhibit increased firing rates compared to lean mice [58]. We therefore propose that GluN2B deletion from AgRP neurons of *Lep*^*ob/ob*^ mice attenuates this hyperactivity and therefore reduces neuropeptide (and neurotransmitter) release, including AgRP. This would then allow for disinhibition of central melanocortin receptor action, ultimately affecting blood glucose levels via a CNS-peripheral pathway that is not yet fully understood [7]. GluN2B deletion thus mimics the anti-diabetic effect of exogenous leptin acting on leptin receptors expressed on AgRP neurons of *Lep*^*ob/ob*^ mice [7,9,59]. These results bring forward the question of why loss of NR2B in AgRP neurons only improves glucose homeostasis in *Lep*^*ob/ob*^ mice but is without effect in lean mice. We speculate that the normal glucose level present in control animals (per definition) is robustly defended by a number of other systems and mechanisms, such that the presumed reduction in AgRP neuronal activity caused by the loss of glutamate action via NMDARs does not cause hypoglycemia. This hypothesis could be explored by employing DREADD methodology; e.g. by inhibiting/activating AgRP neurons in normoglycemic wild-type mice and diabetic *Lep*^*ob/ob*^ mice.

NMDARs are expressed within or in close proximity to the post-synaptic density of most excitatory synapses [17,18]. We have recently reported that the leptin receptor, LepRb is also localized to dendrites of hypothalamic POMC and AgRP neurons [60]. This subcellular evidence, together with the *in vivo* metabolic results from the current study, provides at least indirect support for possible cross-talk between LepRbs and NMDARs in dendritic structures. Some evidence is consistent with this notion. For example, leptin can modulate NMDAR channel activity in isolated hippocampal neuronal cultures and brain slices, requiring activation of phosphoinositide 3-kinase, mitogen-activated protein kinase, and Src tyrosine kinase pathways [61,62]. Furthermore, anti-depressant effects of leptin are reported to involve NMDA receptor activity in hippocampal neurons [63,64]. In addition, leptin can affect GluN2B-NMDA receptor-mediated Ca^2+^ influx in cerebellar granule cells via a mitogen-activated protein kinase-dependent pathway [65]. To explain this cross-talk, one possibility worthy of consideration is Src-kinase dependent tyrosine phosphorylation of the intracellular C-terminal domain of GluN2B, which can affect NMDAR channel activity and synaptic surface-localization [66,67]. Indeed, leptin receptors can activate Src family members [68]. Future studies will be needed to elucidate the precise signaling mechanism whereby this potentially critical dendritic cross-talk between leptin receptors and NMDARs may occur.

In addition to mediating acute excitatory neurotransmission, NMDARs are essential mediators of neuronal development, cell survival and synaptic plasticity [21,34,35,69,70]. Indeed, GluN1 deletion from AgRP neurons causes a decrease in dendritic spine number that is associated with an impaired response to fasting [14]. We show here that genetic loss of GluN2B in AgRP neurons leads to a reduction in AgRP cell number and in dendritic length. Although difficult to prove directly, it seems plausible that these structural changes following GluN2B deletion contribute to the metabolic phenotypes observed here; e.g. the attenuated number of orexigenic AgRP neurons may well explain the reduced caloric intake and body weight of GluN2B-AgRP KO mice.

In conclusion, GluN2B expression in hypothalamic AgRP neurons is required for normal control of caloric intake and body weight in lean animals and can influence leptin sensitivity and glucose balance under obese and diabetic conditions. GluN2B containing NMDARs may play a role in AgRP neuronal survival and in excitability/connectivity via changes in dendritic morphology. The latter could potentially provide a mechanism explaining different body weight “set-points” in obesity.
